# Differences in visual field loss pattern when transitioning from SITA standard to SITA faster

**DOI:** 10.1038/s41598-022-11044-8

**Published:** 2022-04-29

**Authors:** Christopher T. Le, Jacob Fiksel, Pradeep Ramulu, Jithin Yohannan

**Affiliations:** 1 Department of Biomedical Engineering, Johns Hopkins University, Baltimore, MD USA; 2 School of Medicine, University of Maryland, Baltimore, MD USA; 3 Department of Biostatistics, Epidemiology, and Informatics, Perelman School of Medicine, Philadelphia, PA USA; 4 Wilmer Eye Institute, Johns Hopkins University, 600 N Wolfe St, Baltimore, MD 21287 USA; 5 Malone Center for Engineering in Healthcare, Johns Hopkins University, Baltimore, MD USA

**Keywords:** Glaucoma, Visual system

## Abstract

Swedish Interactive Threshold Algorithm (SITA) Faster is the most recent and fastest testing algorithm for the evaluation of Humphrey visual fields (VF). However, existing evidence suggests that there are some differences in global measures of VF loss in eyes transitioning from SITA Standard to the newer SITA Faster. These differences may be relevant, especially in glaucoma, where VF changes over time influence clinical decisions around treatment. Furthermore, characterization of differences in localizable VF loss patterns between algorithms, rather than global summary measures, can be important for clinician interpretation when transitioning testing strategies. In this study, we determined the effect of transitioning from SITA Standard to SITA Faster on VF loss patterns in glaucomatous eyes undergoing longitudinal VF testing in a real-world clinical setting. Archetypal analysis was used to derive composition weights of 16 clinically relevant VF patterns (i.e., archetypes (AT)) from patient VFs. We found switching from SITA Standard to SITA Faster was associated with less preservation of VF loss (i.e., abnormal AT 2–4, 6–9, 11, 13, 14) relative to successive SITA Standard exams (*P* value < 0.01) and was associated with relatively greater preservation of AT 1, the normal VF (*P* value < 0.01). Eyes that transition from SITA Standard to SITA Faster in a real-world clinical setting have an increased likelihood of preserving patterns reflecting a normal VF and lower tendency to preserve patterns reflecting abnormal VF as compared to consecutive SITA Standard exams in the same eye.

## Introduction

Visual field (VF) testing is an important modality for the diagnosis and monitoring of glaucoma, a leading cause of irreversible blindness globally^1^. Among the several methods of testing VF loss in clinical practice, the most widely used is standard automated perimetry with the Humphrey Field Analyzer (HFA; Carl Zeiss Meditec). Over time, the testing strategies implemented by the HFA instrument to evaluate VF loss have been refined through multiple iterations, seeking to alleviate the burden of frequent indicated testing^2–4^ and to bridge the gap between recommended guidelines and clinical practice^5–7^. These updates have increased the speed of exams while preserving accuracy and reproducibility. Developed in 1998, the current predominant testing strategy, the Swedish Interactive Thresholding Algorithm (SITA), is available as both SITA Standard, which completes an exam in approximately 7 min per eye, and SITA Fast, which takes approximately 4 min per eye^8,9^. More recently, Heijl et al. developed SITA Faster, a newer strategy derived from SITA Fast, and demonstrated similar testing variability as SITA Standard and a mean testing duration of 2.9 min^10^.

For the SITA Faster testing strategy to successfully translate into more patients meeting recommended testing guidelines through shorter testing durations, studies investigating VF performance when SITA Faster is clinically implemented are necessary. Although Heijl et al. demonstrated that SITA Faster had similar performance to SITA Standard at the same visit, other cross-sectional studies have shown varying agreement between SITA Standard and SITA Faster in mean deviation (MD) and pattern standard deviation (PSD)^11–13^. Furthermore, how VF loss is characterized when one switches from SITA Standard to SITA Faster during a longitudinal follow-up measurement has not yet fully been established. This scenario is especially important to consider as it will allow providers to contextualize and interpret changes in a new SITA Faster test taken by patients who only have prior SITA Standard tests. One previous study has demonstrated that there were differences in the change in mean deviation (ΔMD) in SITA Faster exams following a SITA Standard exam (Standard-Faster sequence) compared to two sequential SITA Standard exams (Standard-Standard sequence)^14^. In this study, VF exams in eyes with moderate and advanced glaucoma, the transition from SITA Standard to SITA Faster was associated with improved VF performance (higher ΔMD). The differences attributable to testing strategy changes could lead to unaware providers underestimating VF progression in these patients after switching to SITA Faster.

This previous work only studied the effect of switching to SITA Faster from SITA Standard strategies on MD. The impact of testing sequence on other measures used to judge VF progression is currently unknown. While MD is a commonly used measure of the global changes of VF loss, metrics to evaluate location-based VF changes are critically important for clinician interpretation as glaucomatous progression is highly location specific. There are several methods to quantify focal VF changes, including the glaucoma hemifield test (GHT), PSD, pointwise linear regression, and archetypal compositional analysis^15–18^. Archetypal compositional analysis in particular has the advantage of decomposing VFs into 16 clinically interpretable patterns of VF loss and independently quantifying each archetype’s (AT) change over time^19,20^. The primary purpose of this investigation is to study the effect of changing VF test strategies from SITA Standard to SITA Faster on the pattern, or AT, of VF loss. To achieve this goal, we explore AT compositional preservation across VF testing strategies, as well as the tendency to transition from abnormal to normal AT compositions, which could lead to the underestimation VF progression. In accordance with previous observations that switching to SITA Faster was associated with an underestimation of VF deficit, we hypothesize that switching to SITA Faster will be associated with stronger normal AT preservation, weaker abnormal AT preservation, and an increased tendency for abnormal AT to predict the normal AT in the subsequent exam relative to consecutive SITA Standard exams.

## Methods

This study protocol was approved by the Johns Hopkins University School of Medicine Institutional Review Board and adhered to the tenets of the Declaration of Helsinki. A waiver of informed consent was obtained by the Johns Hopkins University School of Medicine Institutional Review Board to review VF data.

### Study participants

This study utilized Humphrey 24-2 VF testing data from a previously described cohort of patients. Briefly, all patients received VF testing in one or both eyes from 2018 to 2020 at the Wilmer Eye Institute Glaucoma Center of Excellence in Baltimore, Maryland^14^. Eligible patients were those with manifest glaucoma or those being followed as glaucoma suspects. As the objective of the study was to assess the reliability and reproducibility of different testing strategy sequences, eyes were not excluded based on reliability metrics for our primary analysis. We included eyes that underwent 3 VF examinations that occurred in the following temporal sequence of testing strategies: (1) SITA Standard; (2) SITA Standard; and (3) SITA Faster. We collected VF data from these three exams from eligible eyes (N = 766) for our analysis.

### Statistical analysis: archetypal decomposition and clinical phenotype grouping

**Figure 1:**
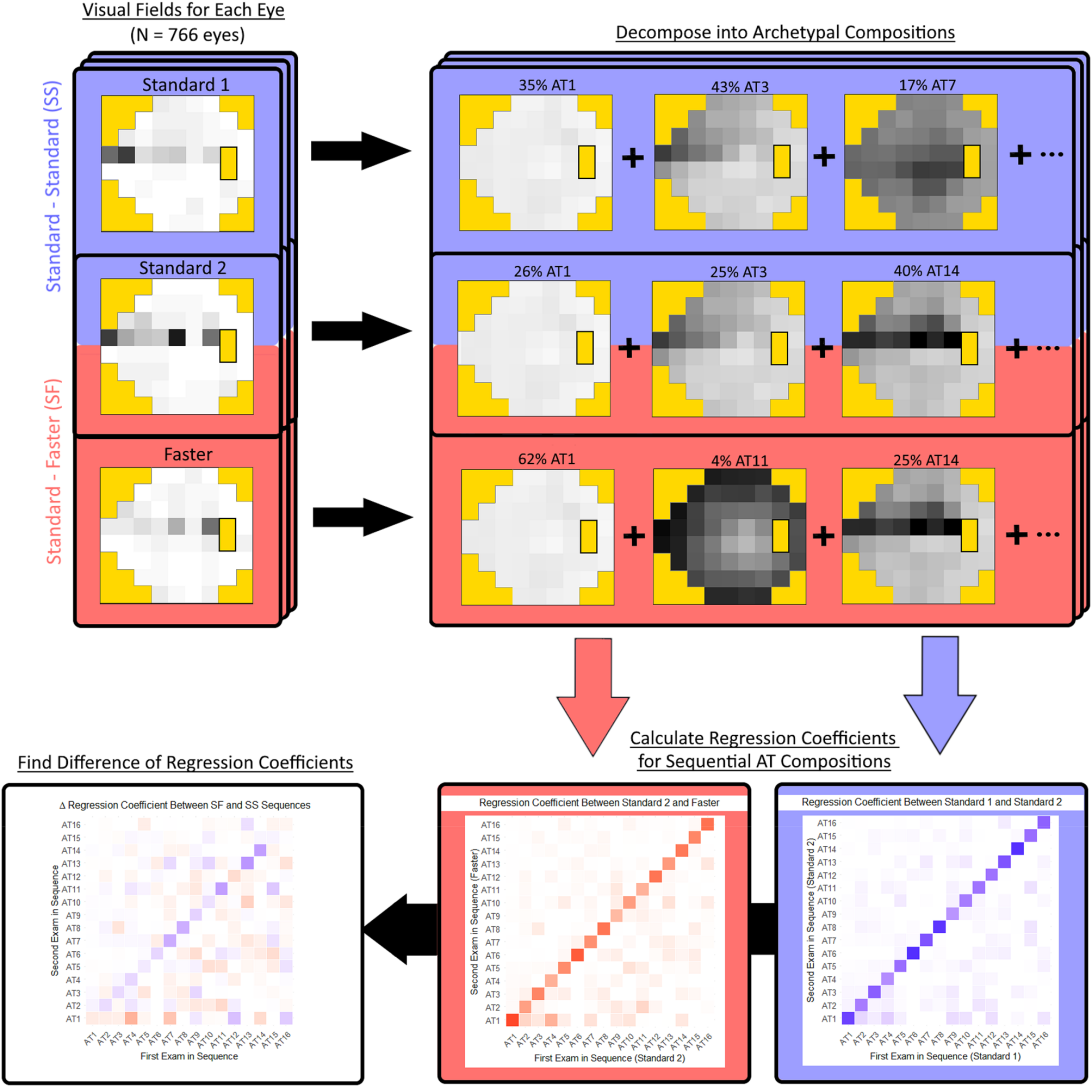
Schematic outlining the methods of the study. Initially visual field data are decomposed into archetypal compositions (top row), the archetypal regression coefficient matrices obtained for Standard-Standard (blue) and Standard-Faster sequences (red) (bottom right). Finally, the regression coefficients are subtracted to yield the final resulting Δ regression coefficients between these two sequences (bottom left).

**Figure 2:**
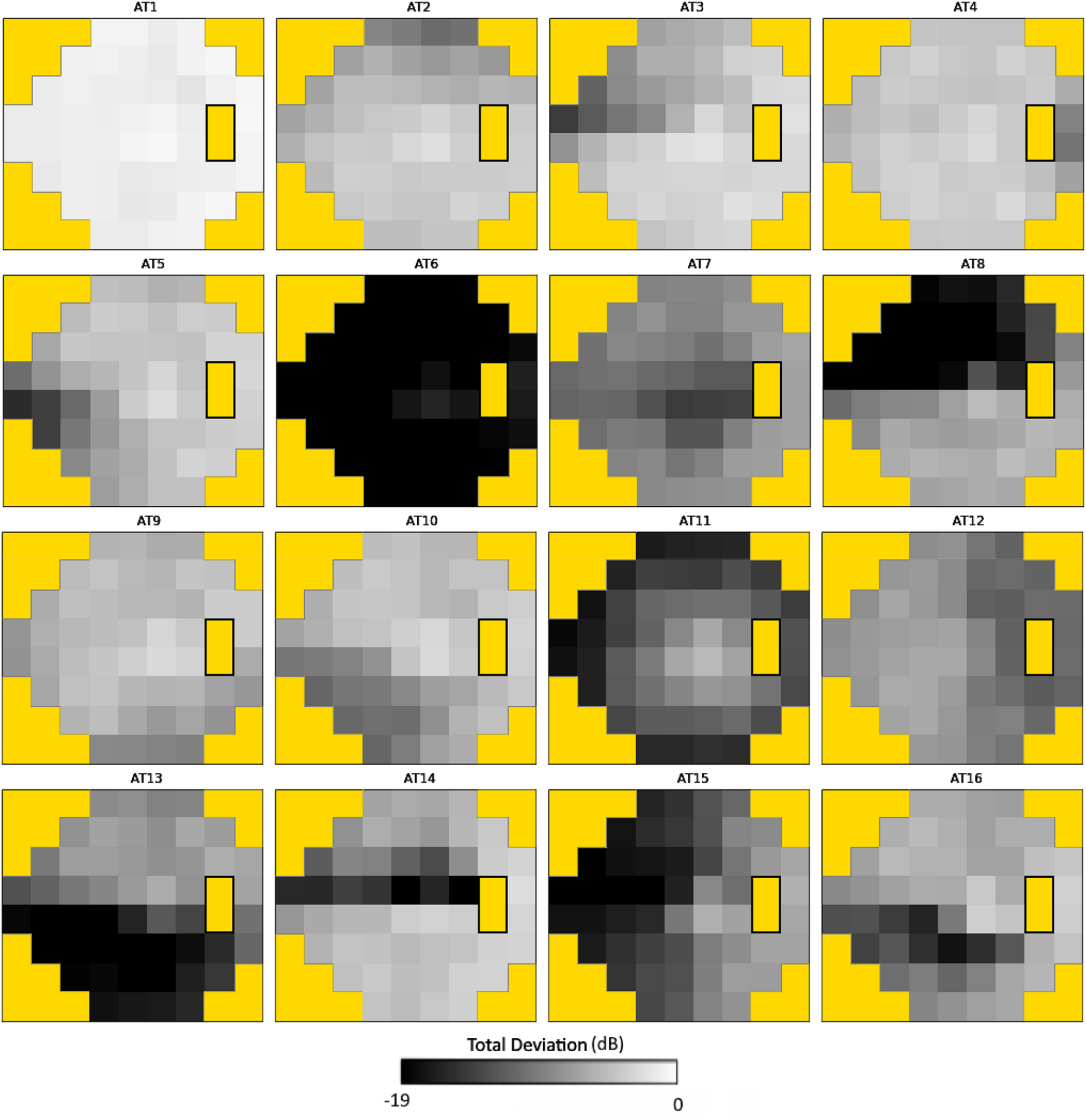
Representation of the 16 Archetypes (AT) in our dataset.

**Figure 3:**
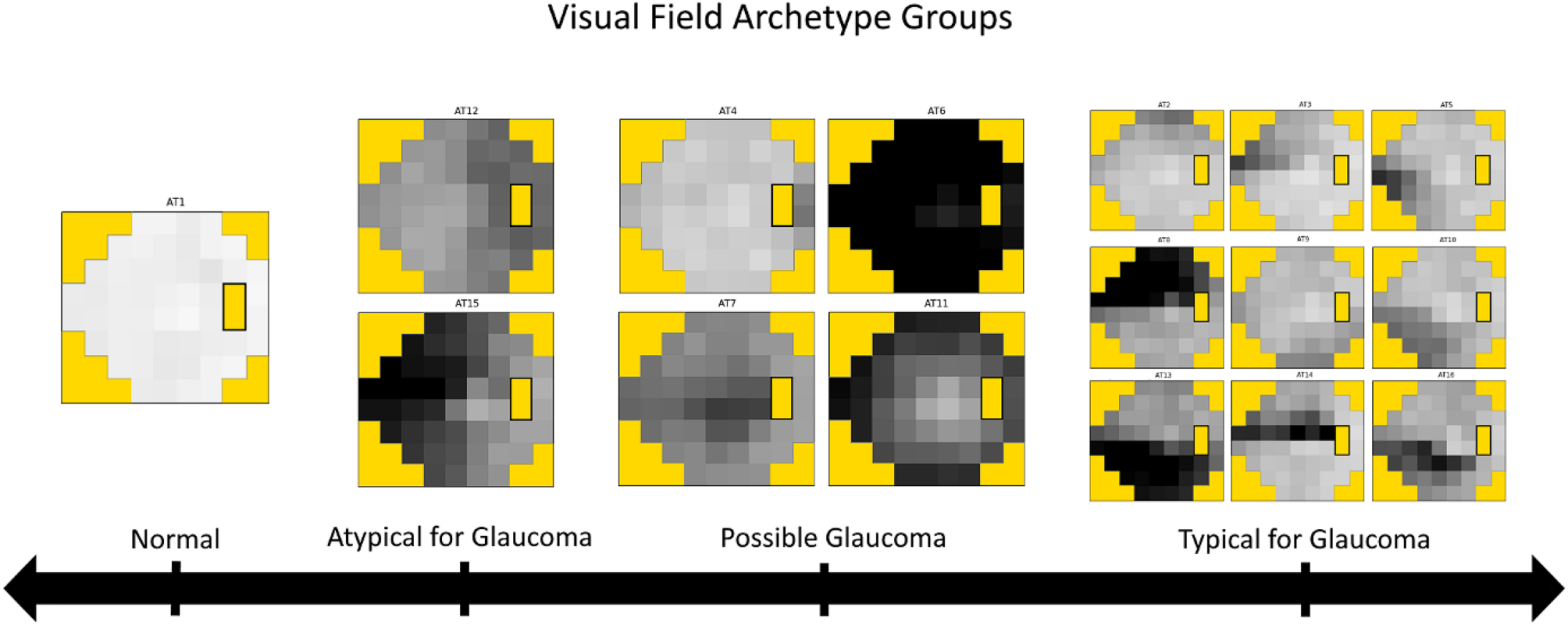
Visual field archetypes grouped by clinical phenotype.

The overall analytic approach is depicted in Fig. 1. Using a previously described VF archetypal decomposition algorithm, we transformed point-wise total deviation values from each VF into non-negative compositional weights summing to 1.00 for 16 clinically interpretable patterns of VF loss^17^. These compositional weights can be thought of as the proportion of each AT that is represented by an individual VF (e.g., % AT 1, % AT 2, etc.). Figure 2 illustrates an expected VF loss pattern for each AT in our dataset that is similar to previously published works^17^. For further analysis exploring clinical phenotype groups, we defined and sorted AT into the following four categories based on pattern phenotypic labels from the work describing VF archetypal decomposition, as well as the observation that VF loss patterns in glaucoma classically respect the horizontal meridian following the course of arcuate nerve fibers^17,21^: normal (AT 1), atypical for glaucoma (AT 12, 15), possible glaucoma (AT 4, 6, 7, 11), and typical for glaucoma (AT 2, 3, 5, 8, 9, 10, 13, 14, 16) (Fig. 3). Patterns aligned to the vertical meridian (i.e., temporal and nasal hemianopia) were considered “atypical for glaucoma”. The patterns classified as “possible glaucoma” (temporal wedge, central scotoma, near total loss, and concentric peripheral defect) can be found in glaucomatous VF but are less commonly found than the altitudinal defects and nasal steps that we classified as “typical for glaucoma”^20,22,23^. The AT compositional weights were summed within each group to derive a compositional weight for the four clinical phenotype groups rather than each of the 16 AT. The groups combine clinically similar phenotypes, establishing broader trends in differences between testing sequences with the benefit of increasing available samples for each regression.

### Linear regression models analysis and δ regression coefficient

We fit a linear regression model between AT composition weights in VF pairs in the Standard-Standard sequences, and AT composition weights in VF pairs in the Standard-Faster sequence (Fig. 1 bottom right)^24^. The 16 by 16 matrix of regression coefficients generated by the model can be interpreted as the association between a first VF exam’s set of 16 AT compositional weights with the second VF exam’s set of 16 AT compositional within each sequence (Standard-Standard or Standard-Faster). Regression coefficient *ij* found in the *i*th column and the *j*th row of the coefficient matrix corresponds to how the compositional weight of AT *i* from the first exam predicts the compositional weight of AT *j* in the second exam. A regression coefficient of one would reflect a very strong association, while a coefficient of zero would reflect a very weak association. In our clinical phenotype grouping analysis (with four clinical phenotypes), the regression coefficient matrix was a 4 by 4 matrix rather than a 16 by 16 matrix. Compared to alternative compositional data regression methods that use log-ratio transformations^25^, this transformation-free modeling method is preferable for our analysis, allowing for a straightforward interpretation of regression between two compositional values and further uniquely permits the existence of some AT to have compositional weights of zero. We subtracted regression coefficients in the Standard-Standard model from the coefficients in the Standard-Faster model to find the difference in these associations for each sequence (Δ regression coefficient) (Fig. 1 bottom left). Positive differences reflect stronger associations in the Standard-Faster model relative to the Standard-Standard model, negative differences reflect the opposite, and differences of zero indicate that there is no difference in association between sequences. We visualized these regression coefficients and their differences using heat maps.

We took particular interest in two sets of Δ regression coefficients: (1) the diagonal of the matrix (“preservation coefficients”), which represents the preservation of AT compositional weights between two exams in the sequence and (2) the AT 1 row of the matrix (“transition-to-normal coefficients”), which represents the transition between all abnormal AT (AT 2–AT 16) to normal archetype (AT 1) between two exams in a sequence. Each of these analyses were performed on regression models incorporating all sixteen AT, as well as models incorporating AT composition weights aggregated by the four clinical phenotype groups. We obtained 99% confidence intervals for Δ regression coefficient matrices using bootstrapping at the patient level to account for inter-eye correlation and used these confidence intervals to establish which differences in coefficients were statistically significant (where the 99% confidence interval does not contain zero). We selected 99% confidence intervals to avoid an inflated Type I error rate due to the numerous statistical comparisons. All statistical analysis was performed with R 4.0.3 (R foundation for Statistical Computing).

## Results

A total of 421 patients with 766 eyes with a glaucoma related diagnosis were included in this study. Descriptive characteristics for the study population and VF characteristics stratified by different testing strategy sequences are shown in Table 1. Of note, 488 (63.7%) eyes had mild or suspect glaucoma, 139 (18.1%) eyes had moderate glaucoma, and 139 (18.1%) eyes had advanced glaucoma when stratified by average MD for each eye across the three exams. Mild, moderate, and advanced disease were defined as average MD better than − 6 decibels (dB), between − 6 and − 12 dB, and less than − 12 dB, respectively. The average time difference between Standard-Standard exams was 440 days (SD = 760 days) and between Standard-Faster exams was 394 days (SD = 592 days) (*P* value = 0.19 by Student’s *t-test*). Supplementary Table 1 shows differences between Standard-Standard exams and Standard-Faster exams based on GHT, pattern deviation (PD), and total deviation (TD) changes. More (19.7% versus 16.6%, *P* < 0.01) mild/suspect eyes transitioning to Standard-Faster changed from “outside normal limits” to “within normal limits” on the GHT compared to the same eyes on Standard-Standard. Of the 704 eyes with PD data available, there was a statistically significantly lower proportion of eyes (14.8% versus 26.4%, *P* < 0.01) with an increase of greater than 15% points with PD probability < 0.01 when transitioning testing strategies in the moderate/advanced eyes. In comparison, there was a statistically non-significantly greater proportion of eyes (11.7% versus 10.2%, *P* = 0.28) of PD points increases in mild/suspect eyes. For increases in TD points, mild/suspect eyes had statistically significantly fewer eyes with greater than 15% change in TD points with TD probability < 0.01 when transitioning than when tested with consecutive SITA Standard exams (8.8% versus 10.2%, *P* = 0.04).

**Table 1:** Demographic and visual field characteristics. *P* values obtained from a paired *t* test (two-tailed).

**Population characteristics**			
Patients	421		
Eyes	766		
Mean Age, years (SD)	69.2 (13.5)		
Severity of Glaucoma			
Mild or Suspect, n eyes (%)	488 (63.7%)		
Moderate, n eyes (%)	139 (18.1%)		
Advanced, n eyes (%)	139 (18.1%)		
**Visual Field Characteristics**			
	**Standard-Standard**	**Standard-Faster**	***P* value**
Difference in MD Between First and Second VF, dB (SD)	− 0.295 (3.42)	0.231 (3.12)	0.01
Difference in PSD Between First and Second VF, dB (SD)	0.158 (1.86)	− 0.369 (2.04)	< 0.01
Time Difference between Tests, days (SD)	439.8 (759.5)	394.4 (592.3)	0.19
Mean MD at Last VF, dB (SD)	− 6.19 (6.94)	− 5.96 (7.03)	0.06
Mean PSD at Last VF, dB (SD)	5.19 (3.82)	4.82 (3.66)	< 0.01
Test Duration at Last VF, s (SD)	377.93 (79.17)	177.77 (52.64)	< 0.01

*SD* standard deviation, *MD* mean deviation, *PSD* pattern standard deviation, *VF* visual field, *dB* decibels, *FP* false positive.

**Figure 4:**
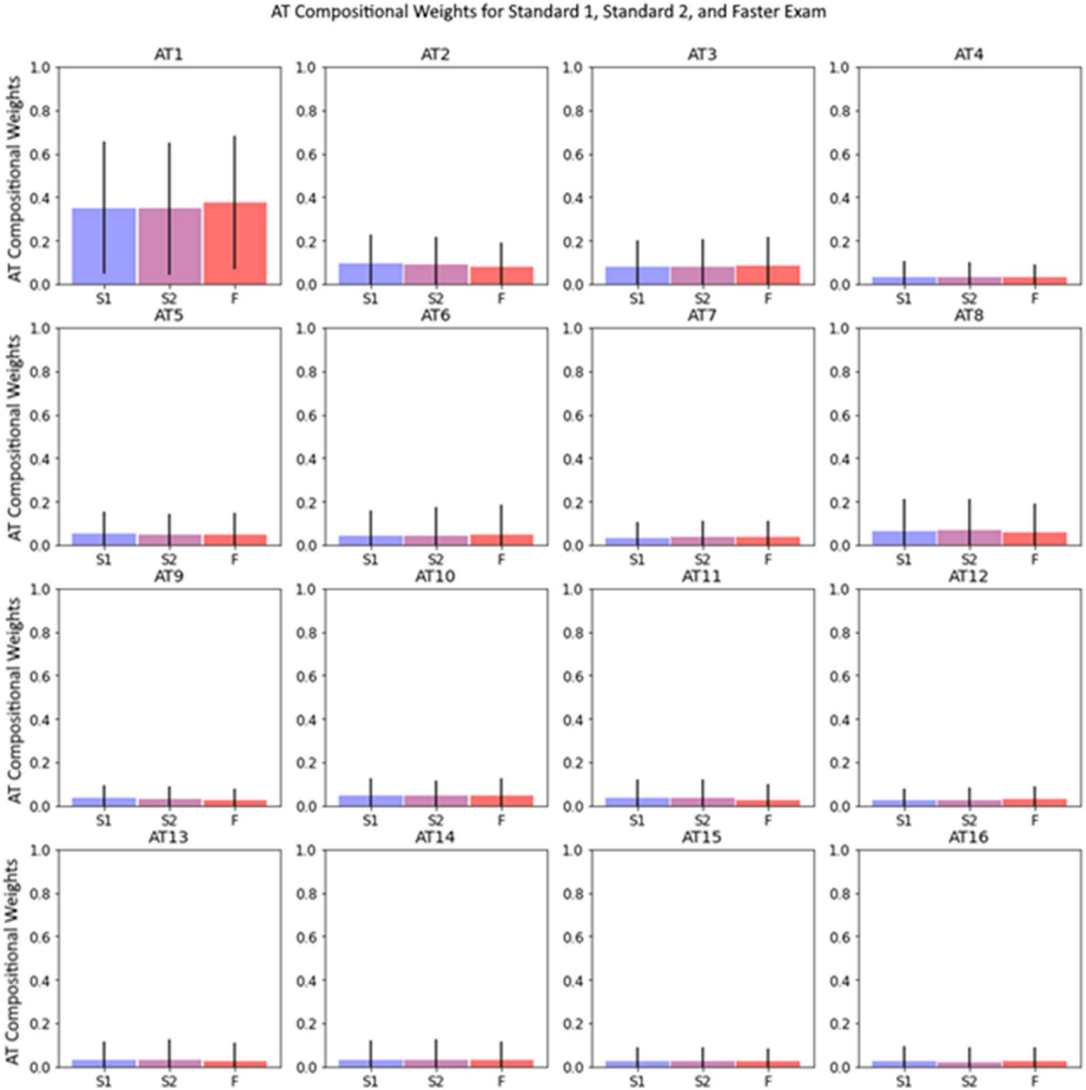
AT 1 (normal VF archetype) had the highest mean AT compositional weight for all exams in the sequence, with statistically non-significantly higher average weights in the SITA Faster exam (0.37 ± 0.30) than the first SITA Standard (S1) and second SITA Standard (S2) exams (0.35 ± 0.30 and 0.35 ± 0.30, respectively) (*P* value = 0.18 by analysis of one-way variance).

The relationship between mean AT compositional weights and the temporal sequence of VF exam are depicted in Fig. 4. AT 1 (normal VF archetype) had the highest mean AT compositional weight for all exams in the sequence, with statistically non-significantly higher average weights in the SITA Faster exam (0.37 ± 0.30) than the first SITA Standard (S1) and second SITA Standard (S2) exams (0.35 ± 0.30 and 0.35 ± 0.30, respectively) (*P* value = 0.18 by analysis of one-way variance).

**Figure 5:**
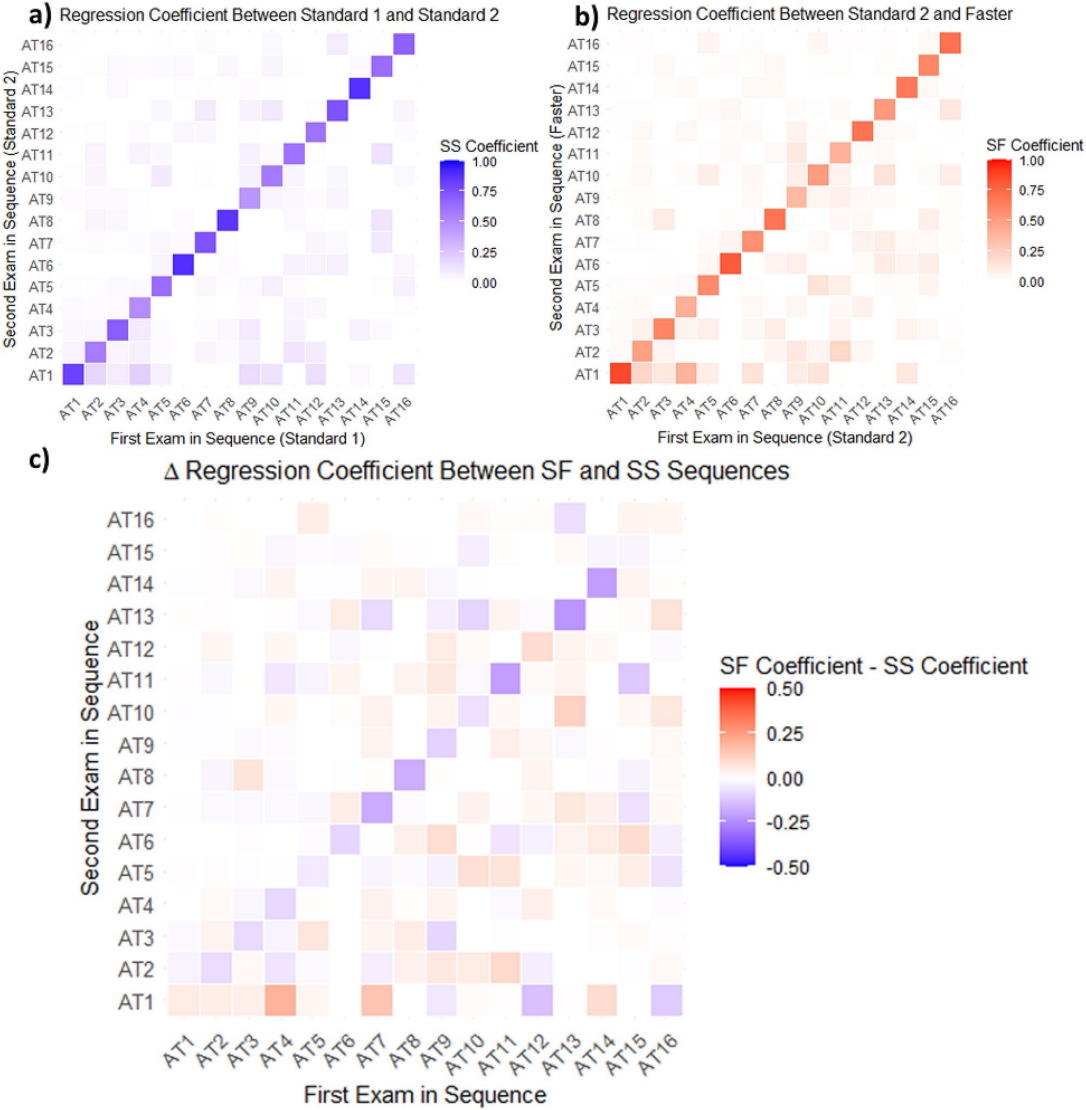
(**a**) Heatmap of archetypal regression coefficients by archetype in Standard-Standard (SS) by archetype (AT). (**b**) Heatmap of archetypal regression coefficients by archetype in Standard-Faster (SF) by AT. (**c**) Heatmap of Δ regression coefficients by AT.

Figure 5a and b show AT regression coefficients between each pair of successive VF exams in the sequence. The largest magnitude regression coefficients are found along the matrix diagonals, with a median of 0.66 and interquartile range (IQR) of 0.60–0.76 for the Standard-Standard sequence and 0.60 (IQR 0.50–0.69) for the Standard-Faster sequence, reflecting the preservation of AT from exam to exam in both sequences. In the off diagonals, the median regression coefficient in the Standard-Standard sequence was 0.006 (IQR 0–0.03) and 0.01 (IQR 0 to 0.04) in the Standard-Faster Sequence. The most preserved AT in the Standard-Standard sequence was AT 6 (near total loss) (0.89), while the most preserved AT in the Standard-Faster sequence was AT 1 (normal) (0.87). The least preserved in both sequences was AT 9 (inferotemporal defect) (0.46 and 0.36 for Standard-Standard and Standard-Faster sequences, respectively).

**Figure 6:**
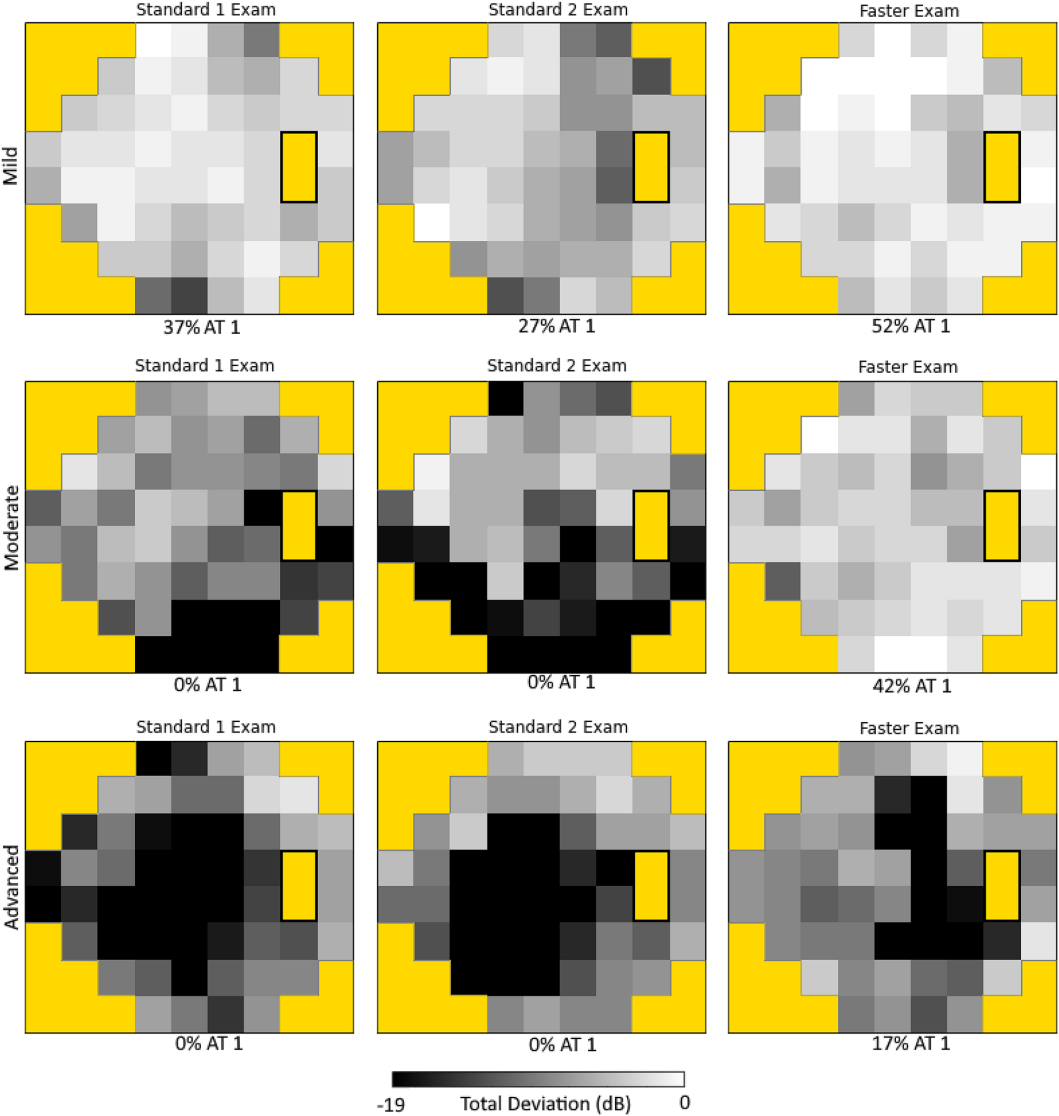
Visual fields from each exam within testing sequences for sample patients with mild, moderate, and advanced disease severity with corresponding AT 1 (normal) AT compositional weights.

A heatmap visualization for the Δ AT regression coefficients found between pairs of successive VF pairs is shown in Fig. 5c with blue tiles indicating higher association between Standard-Standard, and red tiles indicating higher association between Standard-Faster. Overall, the values along the diagonal have higher association in Standard-Standard with an exception noted in AT 1 (normal). These Δ preservation coefficients represent that the Standard-Faster sequence shows relatively weaker preservation in more abnormal AT (representing various patterns of VF loss), but stronger preservation for AT 1 (representing a normal VF) compared to the Standard-Standard sequence. The values along the AT 1 (normal) row have higher associations in Standard-Faster, reflecting the tendency for an abnormal AT in the first exam (SITA Standard) to transition from abnormal to normal in the second exam (SITA Faster), except for AT 9 (inferotemporal defect), AT 12 (temporal hemianopia), and AT 16 (inferior paracentral defect). Table 2 shows the Δ preservation coefficients and Δ transition-to-normal regression coefficient values and 99% confidence intervals between the Standard-Standard and Standard-Faster VF pairs. There were statistically significant differences noted in preservation coefficients for all AT except AT 5 (inferonasal step), 10 (inferonasal defect), 12 (temporal hemianopia), 15 (nasal hemianopia), and 16 (inferior paracentral defect). All statistically significant Δ preservation coefficients (along the diagonal in Fig. 5c) were negative, indicating stronger archetype preservation in the Standard-Standard sequence, except those for AT 1 (normal) (0.05 (99% CI: 0.03–0.08)), indicating better preservation in the Standard-Faster sequence for the normal VF archetype. All statistically significant Δ transition-to-normal regression coefficients (along the bottom row in Fig. 5c) were positive, indicating that there was a higher likelihood for abnormal ATs to transition to normal when switching from SITA Standard to SITA Faster exams. Figure 6 depicts sample individuals’ visual fields from each exam in the testing sequence along with corresponding AT 1 (normal) compositional weight.

**Table 2:** Influence of sequence on Δ preservation coefficient and Δ transition-to-normal regression coefficient by archetype. Positive Δ regression coefficients reflect stronger associations in when transitioning from SITA Standard to SITA Faster compared to consecutive SITA Standard exams, while negative Δ regression coefficients reflect weaker associations.

Archetype # (Pattern Label)	Δ preservation coefficient *P value < 0.01	Δ transition-to-normal coefficient *P value < 0.01
1 (Normal)	0.051 (0.029, 0.076)*	
2 (Superior Peripheral Defect)	− **0.074 (**− **0.140, **− 0.010)*	0.045 (− 0.039, 0.131)
3 (Superonasal Step)	− **0.078 (**− **0.159, **− 0.003)*	0.042 (− 0.048, 0.111)
4 (Temporal Wedge)	− **0.084 (**− **0.175, **− 0.009)*	0.196 (0.016, 0.372)*
5 (Inferonasal Step)	− 0.045 (− 0.118, 0.050)	0.024 (− 0.073, 0.154)
6 (Near Total Loss)	− **0.087 (**− **0.166, **− 0.021)*	− 0.000 (− 0.000, 0.000)
7 (Central Scotoma)	− **0.179 (**− **0.251, **− 0.088)*	0.151 (0.055, 0.209)*
8 (Superior Altitudinal Defect)	− **0.175 (**− **0.228, **− 0.099)*	− 0.000 (− 0.000, 0.000)
9 (Inferotemporal Defect)	− **0.096 (**− **0.158, **− 0.008)*	− 0.050 (− 0.215, 0.113)
10 (Inferonasal Defect)	− 0.064 (− 0.143, 0.004)	0.012 (− 0.156, 0.144)
11 (Concentric Peripheral Defect)	− **0.208 (**− **0.304, **− 0.136)*	0.007 (− 0.039, 0.020)
12 (Temporal Hemianopia)	0.088 (− 0.038, 0.207)	− 0.135 (− 0.195, 0.038)
13 (Inferior Altitudinal Defect)	− **0.226 (**− **0.336, **− 0.145)*	0.002 (− 0.000, 0.030)
14 (Superior Paracentral Defect)	− **0.208 (**− **0.298, **− 0.098)*	0.092 (− 0.021, 0.158)
15 (Nasal Hemianopia)	− 0.022 (− 0.130, 0.079)	− 0.000 (− 0.000, 0.000)
16 (Inferior Paracentral Defect)	0.024 (− 0.091, 0.131)	− 0.110 (− 0.208, 0.000)

Statistically significant values (*P* value < 0.01) are in bold.

**Figure 7:**
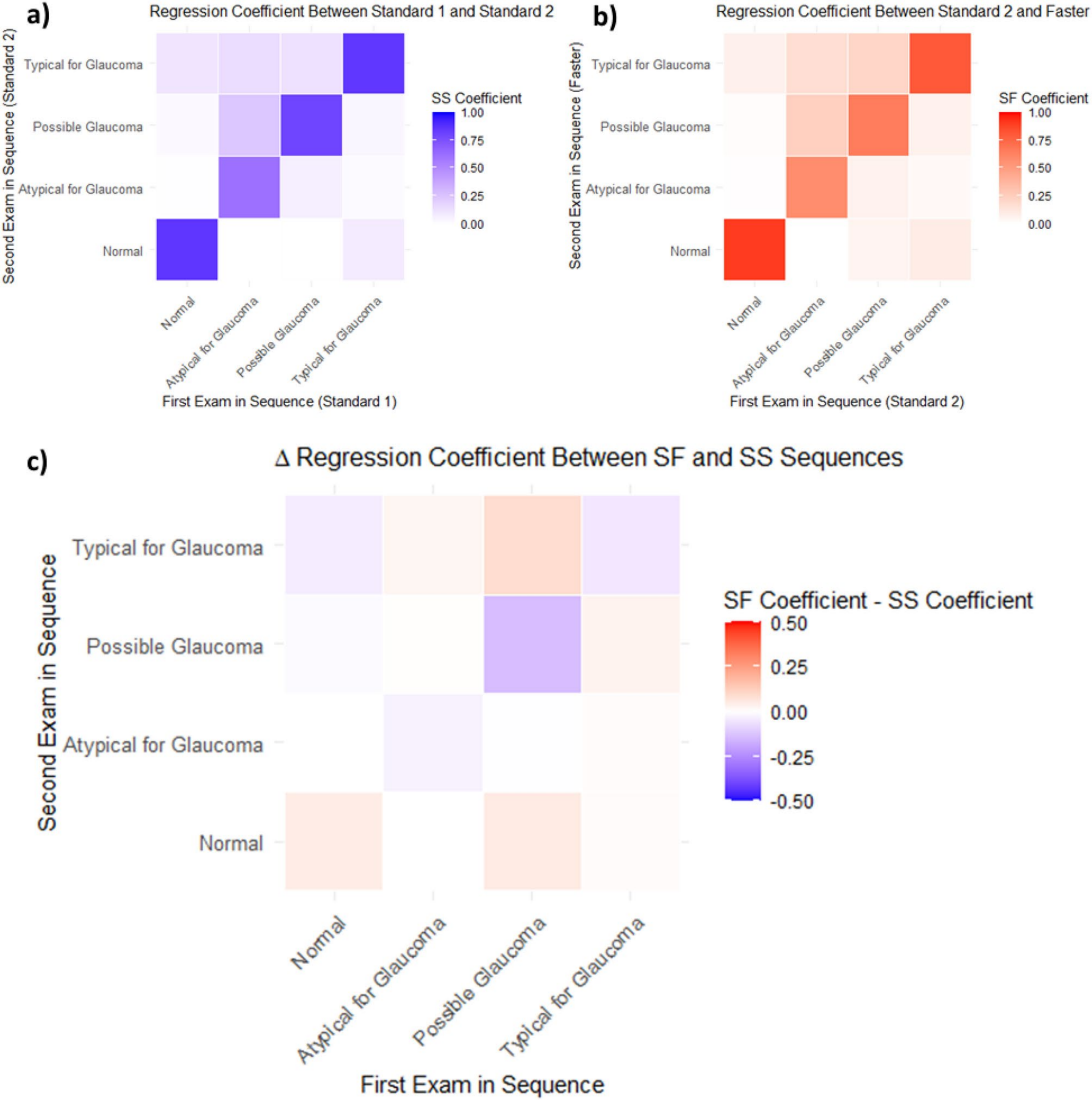
(**a**) Heatmap of archetypal regression coefficients by archetype in Standard-Standard (SS) by clinical phenotype group. (**b**) Heatmap of archetypal regression coefficients by archetype in Standard-Faster (SF) by clinical phenotype group. (**c**) Heatmap of Δ regression coefficients by clinical phenotype group.

When ATs are grouped into clinical phenotypes and analyzed, the regression coefficients for each pair of VFs in the sequence (Fig. 7a and b) and their differences (Fig. 7c) again demonstrate strongest coefficients along the diagonal, with Standard-Standard VF pairs showing stronger preservation coefficients in abnormal phenotypes (VF loss) and a weaker preservation coefficient for the normal phenotype. The largest magnitude regression coefficients are found along the matrix diagonals, with a median value of 0.83 (IQR 0.76–0.86) for the Standard-Standard sequence and 0.73 (IQR 0.64–0.83) for the Standard-Faster sequence, reflecting the preservation of clinical phenotype groups from exam to exam in both sequences. In the off diagonals, the median regression coefficient in the Standard-Standard sequence was 0.05 (IQR 0.02–0.11) and 0.07 (IQR 0.03 to 0.11) in the Standard-Faster Sequence. The most preserved phenotype group for both the Standard-Standard and the Standard-Faster sequence was normal (0.86 and 0.91, respectively). The least preserved clinical phenotype group in both sequences was the group of ATs considered atypical for glaucoma (0.61 and 0.58 for Standard-Standard and Standard-Faster sequences, respectively).

Table 3 shows the Δ preservation coefficients and transition-to-normal regression coefficients for clinically grouped ATs across the pairs of VF tests in the Standard-Standard and Standard-Faster sequences. There were statistically significant differences in the preservation coefficients between VF pairs in the testing sequences for all phenotype groups, except the “atypical for glaucoma” phenotype group. Both possible glaucoma and typical glaucoma clinical phenotypes had negative Δ preservation coefficients (along the diagonal in Fig. 7c), while the normal clinical phenotype had a positive Δ preservation coefficient (0.05 [99% CI: 0.03–0.07]), indicating that the possible glaucoma and typical for glaucoma clinical phenotypes were less preserved within the Standard-Faster pair of VFs, while the normal phenotype was more preserved relative to Standard-Standard pair of VFs. Regarding the Δ transition-to-normal regression coefficients (along the bottom row in Fig. 7c), there was a trend toward transition from abnormal phenotype groups toward normal phenotype grounds in the Standard-Faster sequence compared to the Standard-Standard sequence (i.e., positive coefficient) but none of the differences reached statistical significance.

**Table 3:** Influence of sequence on Δ preservation coefficient and Δ transition-to-normal regression coefficient by clinical phenotype group. Positive Δ regression coefficients reflect stronger associations in when transitioning from SITA Standard to SITA Faster compared to consecutive SITA Standard exams, while negative Δ regression coefficients reflect weaker associations.

Clinical phenotype	Δ preservation coefficient *P value < 0.01	Δ transition-to-normal phenotype *P value < 0.01
Normal	0.048 (0.025, 0.073)*	
Atypical for Glaucoma	− 0.028 (− 0.091, 0.073)	0.000 (− 0.031, 0.000)
Possible Glaucoma	− **0.141 (**− **0.187, **− 0.090)*	0.053 (− 0.001, 0.079)
Typical for Glaucoma	− **0.050 (**− **0.078, **− 0.025)*	0.010 (− 0.009, 0.037)

Statistically significant values (*P* value < 0.01) are in bold.

We performed further sensitivity analyses to investigate the impact of VF reliability on our findings regarding differences in AT preservation and transition-to-normal AT between Standard-Standard and Standard-Faster VF pairs. In a cohort of eyes (n = 611) that met previously established reliability criteria^26^ in all VF exams (a false-positive error rate less than 15% and had a and false negative errors ≤ 25% or ≤ 50% for VFs with MD of > − 6 dB and ≤ − 6 dB, respectively), we found fewer statistically significant effects, but overall similar trends with our primary analysis (Supplementary Tables 2 and 3). Specifically, for all statistically significant Δ preservation coefficients in the primary analysis, there was weaker preservation of abnormal AT and phenotype groups, but stronger preservation in the AT 1 (normal VF) and normal phenotype group in Standard-Faster VF pairs relative to Standard-Standard VF pairs. All statistically significant differences in transition-to-normal coefficients were positive, similarly indicating a greater tendency to transition from abnormal-to-normal AT and phenotype groups in Standard-Faster sequences. We also looked at the reliable fields in a cohort of mild only (n = 371) versus moderate and advanced eyes only (n = 240) to further evaluate the impact of disease severity (Supplementary Tables 4 and 5). Both groups featured statistically significantly weaker preservation of the abnormal AT and phenotype groups in the Standard-Faster sequence. In mild eyes, there was preservation of the normal AT was statistically significantly stronger; while in moderate/advanced there was a statistically non-significant stronger preservation in Standard-Faster sequences compared to Standard-Standard sequences.

## Discussion

While several previous studies have looked at the accuracy of overall measures of VF damage across different types of VF testing algorithms, including the newer SITA Faster algorithm, to our knowledge, this is the first study to explore the effect of transitioning testing strategies on localized patterns of VF loss. In this work, we have found that eyes that transition from SITA Standard to SITA Faster in a real-world clinical setting have an increased likelihood of preserving patterns reflecting a normal VF and lower tendency to preserve patterns reflecting abnormal VF relative to consecutive Standard exams in the same eye. This finding indicates that clinicians may see some change in VF loss pattern when altering testing algorithms, with a slightly greater move towards a normal visual field pattern when transitioning from SITA Standard testing to SITA Faster testing.

In our previous study, we had found that MD, a global measure of VF loss, improved in eyes transitioning from SITA Standard to SITA Faster compared to consecutive SITA Standard exams^14^. In this study, we found a similar tendency to underestimate longitudinal VF loss or correct for previously overestimated VF loss as demonstrated by decreased preservation of VF loss patterns relative to consecutive SITA Standard exams. While no other studies have looked at differences in VF patterns across exam strategies, other works have explored other measures of focal VF loss, such as PSD. Lavanya et al. found statistically significantly improved PSD (4.7 dB in SITA Faster versus 4.8 dB in SITA Standard, *P* value = 0.01) and nasal threshold values (26 dB in SITA Faster versus 25 dB in SITA Standard) in eyes with SITA Faster strategy exams compared to SITA Standard strategy exams from the same day in a cohort of 97 eyes with varying glaucoma status^13^. While these differences were statistically significant, the authors did not consider these effects clinically significant. Additionally, all other sectors apart from nasal were not statistically different between the testing strategies. In another study, Phu et al. found statistically non-significant differences in PSD between SITA Faster and SITA Standard strategy examinations performed on the same day; however, the authors note that there is an overestimation of sensitivity in SITA Faster exams that manifests as an underestimation of the probability score on the PD plot^11^. These findings led the authors to conclude that SITA Faster and SITA Standard were not necessarily interchangeable, particularly in advanced disease, but needed further studies to understand the clinical ramifications in long-term monitoring. Heijl et al. similarly found that the number of test points depressed at the *P* < 0.01 level in the PD plot was statistically significantly larger in SITA Standard than in SITA Faster with a mean difference of 1.1 PD points^10^. These results demonstrate a similar trend of the SITA Faster strategy underpredicting focal VF loss compared to SITA Standard, or SITA Standard overpredicting focal VF loss relative to SITA Faster. This trend is also seen in our results where we observe statistically significantly higher mean PSD in the second SITA Standard exam than in the SITA Faster exam, as well as an average decrease in PSD (− 0.369 ± 2.04 (s.d.) dB) when transitioning from SITA Standard to SITA Faster compared to an average increase (0.158 ± 0.186 (s.d.) dB) in the Standard-Standard sequence. Additionally, the proportion of eyes with at least a 15% increase in the number of points with a PD probability < 0.01 was 12.6% when transitioning from SITA Standard to SITA Faster compared to 15.2% in consecutive SITA Standard exams. In the same study, Heijl et al. found that SITA Fast and SITA Faster had a mean difference of 0.2 PD points at the *P* < 0.01 level. In a work by Budenz et al., SITA Fast was reported to have a PSD difference of 0.2 dB less than SITA Standard in normal eyes and 0.4 dB less than SITA Standard in eyes with glaucoma^27^. While Bundenz’s work focused on comparisons of 30–2 Full Threshold testing with 30–2 SITA Standard and SITA Fast, the similar performance of SITA Fast and SITA Faster contextualize our results as potentially extending studies comparing SITA Standard and SITA Fast to similar relationships between SITA Standard and SITA Faster. Ultimately, the PSD and PD probability plots results from these prior studies are not entirely conclusive and sometimes differ with one another. In contrast to these studies, our investigation has several additional strengths that may account for clear differences in our results. Firstly, our study features a larger sample size of glaucoma suspect and glaucoma patients. The studies by Lavanya et al., Phu et al., and Heijl et al. included 89, 196, and 126 eyes, respectively, from glaucoma suspects or manifest glaucoma subjects, while our study includes 766 eyes. This difference may improve our statistical power to detect a difference between strategies; although, we note there are differences between disease severity and VF reliability criteria in our studies compared to these studies. Secondly, our work compares the effect of transitioning from SITA Standard to SITA Faster at routine follow-up (several months to about a year) rather than same-day examination, which has been used in all prior studies, and is more representative of real-world conditions. Finally, while PD probability plots and PSD do reflect focal depression or VF sensitivity loss, these metrics have limitations as they simply represent deviation from a uniform loss and do not provide any information regarding a clinically interpretable pattern of VF loss. The PSD for two distinct AT can be very similar, despite having different implications. For example, PSD in advancing glaucoma may improve due to global depression. Our study uses AT compositions, which would effectively capture associations in transitioning between AT 1 (normal) to AT 6 (near total loss) in this example. The precision of AT compositions in describing clinical patterns of VF loss may explain the consistency of our observations relative to those from studies that report more general summary metrics like PSD and PD plots.

The underlying reasons for the differences in focal pattern loss remain unclear. With no way of extracting the ground-truth for these cases, the observed differences in our study can be explained as some combination of two potential factors: the correction of any overestimated VF loss in SITA Standard, as well as design differences contributing to an underestimation of VF loss in SITA Faster. Visual fatigue-related artifact may be why transitioning to SITA Faster is associated with stronger preservation of the AT 1 and weaker preservation of abnormal AT compared to the consecutive SITA Standard exams. There is existing evidence, based on comparisons between Full Threshold and SITA algorithms, that faster VF exams result in higher sensitivity during perimetric testing^28–31^. This difference is most likely due to greater visual fatigue associated with longer testing duration^32^. In our data set, SITA Standard exams had a mean test duration of 376 ± 79 (s.d.) seconds and SITA Faster exams had a mean test duration of 178 ± 53 (s.d.) seconds. By extension, it is reasonable that less visual fatigue during a SITA Faster exam would address any fatigue-related VF loss artifact (and thereby result in more normal pattern of VF changes as seen in AT 1) compared to a preceding SITA Standard exam of longer duration. These differences would inflate the presence of abnormal AT in the Standard-Standard sequence compared with the Standard-Faster sequence and would potentially even lead to some abnormal AT to be associated with a transition to AT 1 (the normal archetype) in the Standard-Faster sequence. In our results, AT 4 (temporal wedge) and AT 7 (central scotoma) patterns, which were both considered “possible glaucoma”, were statistically significantly associated with a stronger tendency to transition to AT 1 (normal) in a SITA Faster exam which could suggest that these patterns of VF loss that are not necessarily associated with glaucoma (i.e., biological changes causing VF loss) tend to be more influenced by visual fatigue/longer test durations.

Another potential contributing to the stronger preservation of AT in SITA Faster is a difference in the assumptions and underlying methodology differences in SITA Faster compared to SITA Standard^10^. SITA Faster starts with age-corrected thresholds at the four primary points during initialization, while SITA Standard begins with 25 dB stimuli. This higher initialization stimulus in SITA Standard could result in a positive start bias, which would promote greater false positives (FP) rates in SITA Faster due to uncertainty during testing. A difference in FP rates between SITA Standard and SITA Faster was present in our data and is consistent with previous studies^10,11,13^. The increased number of FPs in SITA Faster may play a role in the results; however, our prior analysis indicated that even after adjusting for FP rates, there was a statistically significant difference in MD between strategies^14^. We are unable to account for FP as a covariate with our compositional regression modeling methods and so in our current analysis, it is unclear if increased FP rates in SITA Faster exam accounts for these effects alone. Another potential methodology difference that would lead to underestimation of VF defects is the error related factor (ERF) calculations in SITA Faster that are based on SITA Fast distributions rather than the Full Threshold distributions used for SITA Standard. The ERF is used in VF testing to determine at which point the algorithm can stop testing a location before completing the full staircase and all reversals. Other studies have suggested that the ERF differences could be an explanation for higher MD values in SITA Faster compared to SITA Standard, particularly in the foveal thresholds^12^. The observation that foveal threshold values were higher in SITA Faster exams than SITA Standard exams complements our findings that AT 7 (central scotoma) was more strongly associated with transition to AT 1 (normal) in the Standard-Faster sequence compared to the Standard-Standard sequence.

It is important to note that although our results demonstrate statistically significant differences between AT compositions between testing sequences, these effects are relatively subtle. The largest magnitude Δ regression coefficient translated to 0.23 weaker preservation of AT 13 (inferior altitudinal defect) when transitioning to SITA Faster than in consecutive SITA Standard exams. There were statistically non-significant differences between the average composition for each AT within each of the strategies, as shown in Fig. 4. In a prior work that compared changes in AT compositional coefficients to existing clinical progression, authors found that thresholds of AT compositional weight changes per year (e.g., 0.005/year for AT 13) were in fair agreement with existing methods of progression analysis and outperformed these methods in agreement with clinician evaluation on a subset of data^17^. The differences found in our study could impact whether these progression thresholds are met. However, the authors of this prior work acknowledge limitations of clinical applicability of these threshold slopes with plans for future iteration and development. Given the relative novelty of archetypal decomposition analysis, practitioners should determine on an individual case basis if the effects of testing sequence on pattern loss are clinically impactful. A reasonable strategy if confronted with pattern differences during transition may be to consider confirmatory testing for further clinical decision making^33^.

When we combined the AT into clinical phenotype groups, we found that trends observed for the overall AT have greater consistency in terms of overall effect and direction. All clinical phenotype groups have an overall stronger tendency to preserve a normal VF pattern when transitioning to SITA Faster and weaker tendency to maintain abnormal patterns of VF loss relative to consecutive SITA Standard exams, particularly in groups associated with possible glaucoma or typical for glaucoma. Although the AT 4 (temporal wedge) and AT 7 (central scotoma) Δ transition-to-normal coefficients were statistically significant in the individual AT analysis and both of these are considered possible glaucoma phenotypes, we found statistically non-significant, but still positive differences in the transition-to-normal coefficient analysis for clinical phenotypes. It is likely that AT 4 (temporal wedge) and AT 7 (central scotoma) are independently associated with a tendency to transition to normal, but not the remaining AT that were considered possible glaucoma, such as AT 6 (total loss).

This study incorporates a large clinical dataset that is likely to capture real-world circumstance compared to previous studies comparing SITA Faster and SITA Standard that use smaller datasets and primarily study short-term shifts in performance between strategy^10^. One limitation of our study is that AT compositional weights are not formally implemented in day-to-day clinical management, and so the clinical impact of our described statistically significant effects are difficult to interpret with certainty. However, AT analysis quantifies changes in clinically interpretable patterns of VF loss and the changes in VF loss pattern (i.e. consistency with retinal nerve fibral layer loss) are relevant for diagnostic and management consideration^4^. Another limitation is potential generalizability of our findings, as this is a single-center study. Further, there is a possibility of selection bias because patient conversion to SITA Faster at this center’s glaucoma clinic was not universal. Future multi-center studies will be needed to investigate the effects of disease severity on patterns of local VF loss in eyes transitioning strategies and to further confirm the generalizability of our findings. Additionally, the time between VF examinations was not held constant for each patient and eye. However, differences between sequences for time between VF examinations was not statistically significant, and the expected change in AT over these time scales in glaucoma patients is overall low^17^. Lastly, and most significantly, there is no clear answer for which VF algorithm (Standard or Faster) represents the ground truth. We report a relative difference between sequences; however, whether the Faster algorithm underestimates or the Standard algorithm overestimates true glaucomatous damage is still to be determined in future work.

The results of this study demonstrate that transition from SITA Standard to SITA Faster is associated with stronger preservation of normal appearing VF patterns and weaker preservation of abnormal VF patterns. This difference may reveal fatigue-related artifact or mask localized disease progression when transitioning strategies, and clinicians should be aware of these differences when interpreting VF exams if they transition patients to the new SITA Faster testing strategy.

## Supplementary Information

Supplementary Information.

## Abbreviations

SITA: Swedish interactive thresholding algorithm
VF: Visual field
AT: Archetype
HFA: Humphery field analyzer
MD: Mean deviation
SD: Standard deviation
SS: Standard-Standard
SF: Standard-Faster
IQR: Interquartile range
CI: Confidence interval
dB: Decibel
PSD: Pattern standard deviation
FP: False positive
GHT: Glaucoma hemifield test
TD: Total deviation
