# Supplementary material for: Differences in visual field loss pattern when transitioning from SITA standard to SITA faster
Source: Sci Rep. 2022 Apr 29;12:7001. doi: 10.1038/s41598-022-11044-8 (PMC9054761; doi:10.1038/s41598-022-11044-8)
Supplement: Supplementary file 1 — Supplementary Information. [file 41598_2022_11044_MOESM1_ESM.pdf]

Differences in Visual Field Loss Pattern When Transitioning from SITA Standard to SITA Faster

Christopher T. Le, MSE, Jacob Fiksel, PhD, Pradeep Ramulu, MD, PhD, Jithin Yohannan, MD, MPH

SUPPLEMENTAL FIGURES

Visual Field Characteristics				
		Standard-Standard	Standard-Faster	P value
Change from Outside Normal Limits by GHT on first VF to Within Normal Limits on second VF (%, n eyes) in Mild or Suspect		16.6% (34/205)	19.7% (43/218)	<0.01
Change from Within Normal Limits by GHT on first VF to Outside Normal Limits on second VF (%, n eyes) in Mild or Suspect		23% (46/200)	21.2% (40/189)	0.65
Increase in >15% significant PD points (P<0.01) between exams	Mild/Suspect %, n	10.2% (50/488)	11.7% (57/488)	0.28
	Moderate/Advanced %, n	26.4% (57/216)	14.8% (32/216)	<0.01
Increase in >15% significant TD points (P<0.01) between exams	Mild/Suspect %, n	10.2% (50/488)	8.8% (43/488)	0.04
	Moderate/Advanced %, n	20.5% (57/278)	15.1% (42/278)	0.30

Supplementary Table 1. Visual field differences based on testing sequence. P values obtained from a paired Tukey's Honest Significance Differences test (two-tailed). (GHT = glaucoma hemifield test, VF = visual field, PD = pattern deviation, TD = total deviation).

**Archetype # (Pattern Label)**	**Δ Preservation Coefficient * P Value < 0.01**	**Δ Transition-to-Normal Coefficient * P Value < 0.01**
1 (Normal)	**0.059 (0.034,0.084)***	
2 (Superior Peripheral Defect)	**-0.089 (-0.171,-0.025)***	0.056 (-0.021,0.165)
3 (Superonasal Step)	-0.043 (-0.154,0.035)	0.031 (-0.072,0.129)
4 (Temporal Wedge)	-0.016 (-0.130,0.068)	0.070 (-0.188,0.244)
5 (Inferonasal Step)	-0.052 (-0.132,0.041)	0.050 (-0.073,0.191)
6 (Near Total Loss)	-0.086 (-0.174,0.001)	0.000 (-0.000,0.000)

7 (Central Scotoma)	**-0.272 (-0.342,-0.146)***	**0.183 (0.054,0.245)***
8 (Superior Altitudinal Defect)	**-0.156 (-0.219,-0.060)***	-0.000 (-0.000,0.000)
9 (Inferotemporal Defect)	-0.056 (-0.112,0.045)	-0.072 (-0.305,0.141)
10 (Inferonasal Defect)	**-0.105 (-0.179,-0.021)***	0.091 (-0.038,0.185)
11 (Concentric Peripheral Defect)	**-0.199 (-0.302,-0.112)***	-0.000 (-0.000,0.000)
12 (Temporal Hemianopia)	0.086 (-0.066,0.235)	-0.104 (-0.182,0.017)
13 (Inferior Altitudinal Defect)	**-0.239 (-0.335,-0.119)***	0.003 (-0.000,0.046)
14 (Superior Paracentral Defect)	-0.117 (-0.222,0.029)	0.082 (-0.041,0.159)
15 (Nasal Hemianopia)	0.069 (-0.109,0.164)	-0.000 (-0.000,0.000)
16 (Inferior Paracentral Defect)	0.028 (-0.091,0.151)	-0.000 (-0.000,0.000)

Supplementary Table 2. Influence of sequence on preservation coefficient and transition-to-normal regression coefficient by archetype for sensitivity analysis in eyes with only reliable visual fields (n=611). Positive Δ regression coefficients reflect stronger associations in when transitioning from SITA Standard to SITA Faster compared to consecutive SITA Standard exams, while negative Δ regression coefficients reflect weaker associations.

Clinical Phenotype	**Δ Preservation Coefficient<br>* P Value < 0.01**	**Δ Transition-to-Normal Phenotype<br>* P Value < 0.01**
Normal	**0.059 (0.032,0.083)***	
Atypical for Glaucoma	-0.006 (-0.116,0.103)	-0.022 (-0.092,0.000)
Possible Glaucoma	**-0.116 (-0.166,-0.036)***	0.044 (-0.000,0.070)
Typical for Glaucoma	**-0.062 (-0.088,-0.035)***	0.019 (-0.001,0.048)

Supplementary Table 3. Influence of sequence on preservation coefficient and transition-to-normal regression coefficient by clinical phenotype group for sensitivity analysis in eyes with only reliable visual fields (n=611). Positive Δ regression coefficients reflect stronger associations in when transitioning from SITA Standard to SITA Faster compared to consecutive SITA Standard exams, while negative Δ regression coefficients reflect weaker associations.

Archetype # (Pattern Label)	Δ Preservation Coefficient * P Value < 0.01		Δ Transition-to-Normal Coefficient * P Value < 0.01	
	Mild Only	Moderate/ Advanced	Mild Only	Moderate/ Advanced
1 (Normal)	**0.062 (0.026,0.089)***	0.096 (-0.128,0.340)		
2 (Superior Peripheral Defect)	**-0.167 (-0.277,-0.080)***	-0.010 (-0.127,0.180)	**0.097 (0.019,0.253)***	0.071 (-0.035,0.152)
3 (Superonasal Step)	-0.140 (-0.252,0.035)	0.084 (-0.057,0.232)	-0.016 (-0.178,0.175)	0.020 (-0.072,0.046)
4 (Temporal Wedge)	-0.035 (-0.167,0.061)	-0.014 (-0.191,0.279)	-0.003 (-0.170,0.250)	-0.000 (-0.051,0.000)
5 (Inferonasal Step)	**-0.181 (-0.316,-0.023)***	0.044 (-0.083,0.251)	0.118 (-0.035,0.304)	-0.058 (-0.141,0.052)
6 (Near Total Loss)	**0.277 (0.011,0.466)***	-0.110 (-0.203,0.010)	0.040 (-0.735,0.383)	-0.000 (-0.000,0.000)
7 (Central Scotoma)	-0.126 (-0.337,0.036)	**-0.236 (-0.359,-0.095)***	0.028 (-0.381,0.301)	**0.178 (0.014,0.239)***
8 (Superior Altitudinal Defect)	0.109 (-0.000,0.278)	**-0.160 (-0.227,-0.039)***	-0.000 (-0.017,0.000)	0.000 (-0.000,0.000)
9 (Inferotemporal Defect)	**-0.161 (-0.247,-0.024)***	-0.027 (-0.183,0.211)	0.163 (-0.209,0.330)	-0.167 (-0.264,0.018)
10 (Inferonasal Defect)	-0.077 (-0.167,0.065)	-0.055 (-0.294,0.058)	-0.092 (-0.317,0.206)	0.133 (-0.059,0.286)
11 (Concentric Peripheral Defect)	**-0.178 (-0.274,-0.012)***	**-0.213 (-0.342,-0.091)***	0.277 (-0.340,0.481)	0.000 (-0.000,0.000)
12 (Temporal Hemianopia)	-0.043 (-0.146,0.050)	0.075 (-0.170,0.355)	-0.247 (-0.463,0.071)	-0.068 (-0.180,0.128)
13 (Inferior Altitudinal Defect)	-0.070 (-0.153,0.025)	**-0.270 (-0.413,-0.088)***	-0.194 (-0.367,0.000)	-0.000 (-0.000,0.045)
14 (Superior Paracentral Defect)	-0.234 (-0.375,0.006)	0.025 (-0.104,0.161)	0.228 (-0.078,0.383)	-0.025 (-0.094,0.076)
15 (Nasal Hemianopia)	0.077 (-0.070,0.177)	0.127 (-0.111,0.257)	0.000 (-0.309,0.236)	-0.000 (-0.000,0.000)
16 (Inferior Paracentral Defect)	0.041 (-0.118,0.329)	0.079 (-0.127,0.217)	-0.092 (-0.413,0.128)	0.000 (-0.004,0.000)

Supplementary Table 4. Influence of sequence on preservation coefficient and transition-to-normal regression coefficient by archetype for mild (n=371), and moderate/advanced eyes (n=240) with only reliable visual fields. Positive Δ regression coefficients reflect stronger associations in when transitioning from SITA Standard to SITA Faster compared to consecutive SITA Standard exams, while negative Δ regression coefficients reflect weaker associations.

Clinical Phenotype	Δ Preservation Coefficient * P Value < 0.01		Δ Transition-to-Normal Phenotype * P Value < 0.01	
	**Mild Only**	**Moderate/ Advanced**	**Mild Only**	**Moderate/ Advanced**
Normal	**0.034 (0.003,0.066)***	0.108 (-0.128,0.333)		
Atypical for Glaucoma	-0.051 (-0.170,0.066)	0.048 (-0.124,0.211)	-0.348 (-0.597,0.036)	0.000 (-0.013,0.034)
Possible Glaucoma	**-0.081 (-0.214,-0.001)***	-0.096 (-0.169,0.025)	0.028 (-0.168,0.281)	0.029 (-0.000,0.046)
Typical for Glaucoma	**-0.085 (-0.138,-0.030)***	**-0.068 (-0.114,-0.018)***	**0.088 (0.036,0.146)***	0.002 (-0.015,0.030)

Supplementary Table 5. Influence of sequence on preservation coefficient and transition-to-normal regression coefficient by clinical phenotype group for mild (n=371), and moderate/advanced eyes (n=240) with only reliable visual fields. Positive Δ regression coefficients reflect stronger associations in when transitioning from SITA Standard to SITA Faster compared to consecutive SITA Standard exams, while negative Δ regression coefficients reflect weaker associations.
